# Nonsurgical Interventions for the Management of Long-Standing Groin Pain in Athletes: A Systematic Review of Randomized Controlled Trials

**DOI:** 10.7759/cureus.40149

**Published:** 2023-06-08

**Authors:** Rui Brito, Patrícia Cruz, Diogo Costa, Sara Afonso, Paula Barros

**Affiliations:** 1 Physical Medicine and Rehabilitation, Centro Hospitalar e Universitário de Santo António, Porto, PRT

**Keywords:** physical therapy, conservative treatment, sports hernia, athletic pubalgia, groin pain

## Abstract

Groin pain is a common problem in athletes, leading to significant distress and long periods of absence from sports. Nonsurgical interventions are usually the first line of treatment. However, the most effective intervention for groin pain is unknown and recommendations are scarce.

The primary objective of this systematic review was to assess the effectiveness of nonsurgical interventions in the treatment of long-standing groin pain in athletes and to provide some guidance for clinical practice and further research.

A search strategy was performed in March 2020 in Pubmed, Google Scholar, PEDro, and Cochrane Central Register of Controlled Trials databases, without any time restrictions. Only randomized controlled trials (RCT) were included for full-text analysis. Data on the patient’s characteristics, duration of pain, study groups, outcome measures results, follow-up time, and return to play time were extracted. The risk of bias in each study was assessed using the Cochrane risk-of-bias assessment tool. Data for analysis could not be pooled for meta-analysis and, as such, a narrative summary of the outcomes was instead performed. The certainty of the evidence was assessed using a variation of the GRADE approach for when a meta-analysis is not possible to perform.

Seven RCTs were included for analysis. Most studies were classified as uncertain risk of bias. All studies provided evidence that nonsurgical interventions have significant positive effects and may lead to good outcomes concerning pain, function, and return to sports at previous levels. The certainty of the evidence was assessed to be low using the modified GRADE approach.

Despite the low quality of the available evidence, nonsurgical treatments demonstrated efficacy in the management of groin pain and should probably be the initial approach to treatment. More RCTs of high quality are necessary to provide clear recommendations on the most efficient nonsurgical treatment strategy for groin pain.

## Introduction and background

Groin pain is a common problem and a significant cause of morbidity in athletes [1]. Pain is usually located in the anterior pelvis which is a complex anatomical structure, causing difficulties in diagnosing the condition [2]. Adding to the problem, there are a variety of terms describing the same pathology (such as athletic pubalgia, sports hernia, Gilmore’s groin, inguinal disruption, etc) [3].

Groin pain is common in sports requiring running, direction changes, and kicking [4]. Imbalances between the abdominal muscles and the hip adductor muscles, combined with excessive loads, may alter the normal distribution of forces in this region leading to pain [1,5]. However, many other factors may be responsible, such as lack of conditioning, poor flexibility, poor posture, and increasing age [5].

Groin pain usually recovers after a period of rest [6]. However, in some cases, the problem persists or recurs over time. Rehabilitation of groin pain usually begins with conservative approaches, which may include physiotherapy and minimally invasive procedures, such as steroid injections. The literature on the best approach to conservative management of groin pain is scarce and of low quality [7]. A previous Cochrane review on this subject included only two randomized controlled trials (RCTs), and none had a low risk of bias. The authors concluded that the available evidence was insufficient to advise on any specific conservative modality [8].

The primary aim of this systematic review was to evaluate the quality of existing evidence to support the use of nonsurgical interventions in the management of long-standing groin pain. To our knowledge, no other published systematic review limited the evaluation to include only randomized controlled trials nor included all forms of non-surgery treatment. The secondary aim was to provide some guidance on treatment recommendations to help guide clinical practice.

## Review

Methods

This systematic review was reported according to the Preferred Reporting Items for Systematic Reviews and Meta-Analyses (PRISMA) guidelines [9]. The clinical question for the systematic review was identified using the PICO (population, intervention, comparison, and outcome) format. Table 1 identifies the categories of the clinical question according to PICO.

**Table 1:** PICO format PICO: population, intervention, comparison, and outcome

PICO	
Population	Athletes with long-standing symptoms of groin pain
Intervention	Conservative care and minimally invasive procedures
Comparison	No treatment, standard physical therapy, and other forms of nonsurgical treatment
Outcomes	Pain scores, return to sports, and functional scales scores

A literature search was performed on PubMed, Google Scholar, PEDro, and Cochrane Central Register of Controlled Trials in April 2023. Only studies published in English were included. There were no time restrictions. The sample search strategy performed on Pubmed consisted of the terms: ((groin pain[MeSH Terms]) OR athletic pubalgia) OR sports hernia[MeSH Terms] AND ((conservative therapy[MeSH Terms]) OR (physical therapy[MeSH Terms])). Studies for inclusion in this systematic review were screened by title and abstract by two reviewers independently. The criteria of eligibility were the following: the study should be an RCT evaluating the efficacy of nonsurgical interventions in the management of long-standing groin pain. A quantitative outcome measure should be reported, such as the reduction in pain, the percentage of athletes returning to sports at the previous level, and the time to recovery. Studies were excluded if other clinical entities besides groin pain were described as a site of origin of pain (such as femoroacetabular impingement). Studies were excluded if patients had any previous surgical treatment performed for groin pain. References of included articles were screened for other studies that might have been missed in the initial search strategy. Data were extracted into a Microsoft Excel sheet (Microsoft, Washington, United States) by two authors. The information collected consisted of the patient characteristics, the diagnosis, duration of pain, type of intervention, study groups, outcome measures, results, follow-up time, and time of return to play.

All forms of nonsurgical care were included in this systematic review such as compression clothing, manual therapy, physical agents use, minimally invasive procedures, and various protocols of exercise. The risk of bias of each RCT was assessed using the Cochrane risk-of-bias assessment tool (Cochrane, London, England) [10]. Data for analysis could not be pooled as there was significant methodological heterogeneity among the studies. As such, a narrative summary of outcomes was performed. The certainty of the evidence was assessed using a variation of the GRADE approach for situations when a meta-analysis cannot be performed and instead, a narrative summary of the effects is provided [11].

Results

Search Results

**Figure 1:**
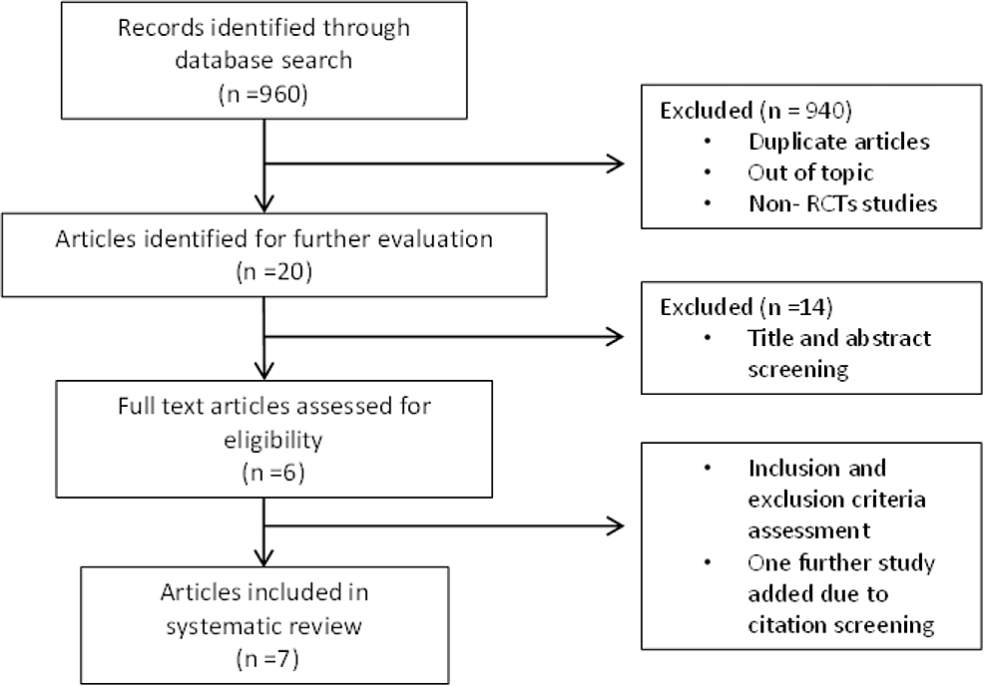
Study search flow diagram

The initial search retrieved 960 studies. After excluding duplicates, limiting the search to include only RCTs, screening the title and abstract, and excluding evidently ineligible articles, only six RCTs were identified that met the inclusion criteria for this systematic review. Citation screening of the included articles revealed one additional relevant article. Figure 1 shows a representation of the study selection process.

Study Characteristics

All of the seven selected studies were randomized controlled trials. Five studies were classified as single-blinded RCTs [12-16] and two were double-blinded RCTs [17,18]. The population size included a total of 345 athletes. The participants consisted mostly of young male adults who practiced sports at a competitive or amateur level, presenting with groin pain for at least a one-month duration. Most of the participants practiced football. All of the studies compared some form of conservative intervention versus a standard treatment approach. The mean age of participants was 28.8 years, the follow-up time after intervention ranged from 12 to 52 weeks, one study did not report follow-up duration, and in one study follow-up was not applicable. Outcome measures were reported in all studies, although primary and secondary outcomes were rarely differentiated. The most common outcome measures were the Visual Analog Scale (VAS) for pain measures, changes in hip range of motion (ROM), the rate of players returning to sport (RTP rate) at previous levels, and the mean time to return to play (RTP time). Two studies used the Numeric Rating Scale (NRS) instead of the VAS as a measure of pain. A few studies also reported changes in functional scales.

A summary of the included studies’ characteristics is presented in Table 2.

**Table 2:** Study characteristics RCT: randomized controlled trial; AT: active training program; PT: physiotherapy; MMT: multi-modal treatment; ET: exercise therapy; VAS: visual analogue scale; ROM: range of motion; RTP: return to sports; NRS: numeric rating scale; PSFS: patient specific functional scale; EPI: intratissue percutaneous electrolysis; ZHC: zoned high compression shorts; NZLC: non-zoned low compression shorts; CS: Copenhagen 5-s squeeze test; IAT: Illinois agility test; ST: maximum shooting; HAGOS: hip and groin outcome score; SW: shock wave therapy; ATP: active therapy program; CT: conventional treatment; RFD: radiofrequency denervation; LAAG: London Adductor and Abdominal Groin score; NA: not applicable

Reference	Study type	N° participants	Mean age	Diagnosis (as described in the study)	Duration of symptoms	Treatment group (N. participants)	Control group (N. Participants)	Follow-up (weeks)	Outcome measures
Holmich et al. (1999) [12]	Single-blinded RCT	68	30 years	Long-standing adductor-related groin pain	9 months (mean duration)	AT program (n =34)	Standard PT (n =34)	24-28 weeks	Treatment success, RTP rate/time, hip ROM
Weir et al. (2011) [13]	Single-blinded RCT	48	28 years	long-standing adductor-related groin pain	8 months (mean duration)	MMT (heat + manual therapy + stretches) (n =26)	ET group (n =22)	16-24 weeks	VAS score during sports, RTP rate/time, hip ROM, objective outcome score (excellent, good, fair, poor)
Moreno et al. (2017) [15]	Single-blinded RCT	24	26 years	Long-standing adductor-related groin pain	At least 1 month. Mean duration not reported	EPI + active PT (n =11)	Active PT (n =13)	24 weeks	Pain reduction measured by the NRS, functional level measured by the PSFS
Otten et al. (2019) [17]	Double-blinded RCT	34	25 years	Long-standing groin pain (adductor, iliopsoas, inguinal, or pubic-related)	At least 1 month. Mean duration not reported	Testing conditions (all participants): wearing ZHC shorts, wearing NZLC shorts	Testing condition (all participants): wearing no compression shorts	NA	Reduction of pain measured by the NRS during the CS, IAT, and ST tests, effect on symptoms measured by HAGOS during actual sport
Schöberl et al. (2017) [18]	Double-Blinded RCT	95	24 years	Groin pain symptoms and MRI diagnosed osteitis pubis	Not provided	2 groups: SWT + intensive physical rehabilitation program (n =26), sham SWT + intensive physical rehabilitation program (n =18)	Absence from sports only (n =51)	52 weeks	Pain reduction measured by VAS, RTP time
Abouelnaga et al. (2019) [14]	Single-Blinded RCT	40	26 years	Sports hernia (athletic pubalgia)	At least 2 months	ATP (n =20)	CT (n =20)	Not reported	Pain reduction measured by VAS, hip ROM
Comin et al. (2013) [16]	Single-blinded RCT	36	43 years	Chronic groin pain without identifiable structural cause	9 months (mean duration)	2 groups: RFD + standard PT-RFD in previous surgery patients (group not randomized) (n =18)	Steroid Injection + standard PT (n =18)	24 weeks	Pain reduction measured with VAS, functional assessment using LAAG score

Summary of Outcomes

A summary of the outcomes of included studies is presented in Table 3. All studies reported positive outcomes of the interventions being tested compared to the respective control groups.

**Table 3:** Summary of outcomes AT: active training program; PT: physiotherapy; MMT: multi-modal treatment; ET: exercise therapy; VAS: visual analogue scale; ROM: range of motion; RTP: return to sports; NRS: numeric rating scale; PSFS: patient-specific functional scale; EPI: intratissue percutaneous electrolysis; ZHC: zoned high compression shorts; NZLC: non-zoned low compression shorts; CS: Copenhagen 5-s squeeze test; IAT: Illinois agility test; ST: maximum shooting; HAGOS: hip and groin outcome score; SWT: shock wave therapy; ATP: active therapy program; CT: conventional treatment; LBP: low back pain; HOOS: hip disability and osteoarthritis outcome score; RFD: radiofrequency denervation; LAAG: London Adductor and Abdominal Groin score

Reference	RTP rate (% / n° patients)	RTP time	Pain scores	Functional scales	Hip ROM	Other outcomes
Holmich et al. (1999) [12]	AT group: 79% (23/29). PT group: 13% (4/30) *P=0.0002	AT group: 18.5 weeks. PT group: not reported	Not reported	None	Hip abduction improved significantly in both groups (p=0.0004). Hip abduction did not show a significant improvement between groups. Adduction strength improved significantly in the AT group compared to PT (p=0.001)	Treatment success (good to excellent results): AT group: 25/34 (74%); PT group: 10/34 (29%) *significant difference in favor of AT (p=0.001). Subjective global assessment: *significant linear trend towards a better effect of AT treatment (p=0.006)
Weir et al. (2011) [13]	MMT group: 50%. ET group: 55% *p =0.78	MMT group: 12.8±6.0 weeks. ET group: 17.3±4.4 weeks *p=0.043	VAS (0-100 score) MMT group: before treatment: VAS=58.9±21.3. After treatment: VAS=36.1±30.1 *p=0.01. ET group: before treatment: VAS=58.5±26.2. After treatment: VAS=21.0±27.0 *p=0.000 ** no significant difference between groups	None	No significant difference after treatment in both groups or between groups (p=0.45, p=0.65)	None
Moreno et al. (2017) [15]	Not assessed	Not assessed	NRS (0-10 scale) EPI+Active PT group: before treatment: NRSpalp: 7.5±1.9; NRScontr: 8.5±1.4. After treatment: NRSpalp: 1.6±1.1; NRScontr: 1.3±0.9. Active PT group: before treatment: NRSpalp:8.1±1.9; NRScontr: 8.0±1.6. After treatment: NRSpalp: 2.5±1.5; NRScontr: 2.2±1.7 *both groups improved pain scores after treatment (p<0.001) **significant difference between groups in NRScontr at all time points (p<0.05) ***significant difference between groups in NRSpalp only at 2 and 4 months follow-up (p<0.01)	PSFS (0-100) EPI + active PT group: Before treatment 55.5±22.2. After treatment 91.6±3.8. Active PT group: before treatment 56.7±20.6. After treatment 87.5±5.6 *PSFS improved significantly in both groups (p<0.001) **PSFS showed no significant difference between groups	Not assessed	Duration of Phase 1 of active PT program was shorter (average 8.8 days) in EPI group (p=0.048)
Otten et al. (2019) [17]	Not assessed	Not assessed	NRS (0-10 score) NRS CS ZHC shorts: 4.6±2.6; NZLC shorts: 5.1±2.4; No compression: 5.4±2.2; NRS IAT ZHC shorts: 2.7±2.4; NZLC shorts: 3.5±2.4; No compression: 4.2±2.5 *significant difference between wearing ZHC and NZLC shorts, small effect **significant difference between wearing ZHC shorts and no compression shorts, medium effect. NRS ST ZHC shorts: 1.8±2.5; NZLC shorts: 2.4±2.2; No compression: 3.0±2.6 *significant difference between wearing ZHC shorts and no compression shorts, small effect. NRS training/matches: NRS during training: ZHC shorts: 4.1±1.9; NZLC shorts: 5.0±2.4; NRS during matches: ZHC shorts: 4.0±2.2; NZLC shorts:5.1±2.5 * significant difference, small effect sizes	None	Not assessed	Wearing ZHC shorts during football significantly improved HAGOS scores on ADL symptoms and sport/recreation subscales (p<0.01, p=0.01, respectively, medium effect sizes)
Schöberl et al. (2017) [18]	42/44 (95%) returned to sports within 4 months (treatment groups)	SW group: 73.2 days (10.5 weeks). Sham SW group: 102.6 days (14.6 weeks). Control group: 240 days (34.3 weeks) *significant difference between groups	VAS (0-10 score) SWT group: before treatment: VAS 8.1±0.8; 1 month after: VAS 3.0±1.4; 3 months after: VAS 0.7±0.6; 1 year after: VAS 0.5±0.5. Sham SWT group: before treatment: VAS 7.8±1.1; 1 month after: VAS 4.6±1.0; 3 months after: VAS 1.7±0.8; 1 year after: VAS 0.7±0.6 *significant improvement in both groups **significant difference between groups at 1 and 3 months	None	Not assessed	Oswestry LBP score showed a significant difference between treatment groups at 1 year favoring SW therapy (p=0.045). HOOS score showed a significant difference between treatment groups at 1 month*, 3 months*, and 1 year** after treatment favoring SW therapy (*p<0.001, **p=0.001). Pain catastrophizing scale showed a significant difference between treatment groups at 3 months favoring SW therapy (p=0.048)
Abouelnaga et al. (2019) [14]	ATP group: 65% (13/20). CT group: 15% (3/20)	Not assessed	VAS (0-10 score) ATP group: before treatment: VAS 7.85±0.74; after treatment: VAS 1.55±068. CT group: before treatment: VAS 7.75±0.71; after treatment: VAS 4.50±060 *significant improvement in both groups (p=0.0001) **significant difference between groups favouring ATP group (p=0.0001)	None	Significant increase in internal and external rotation after treatment in both groups (p=0.0001). No significant difference in internal and external rotation between groups after treatment	None
Comin et al. (2013) [16]	Not assessed	Not assessed	VAS (0-10 score) RFD + standard PT group: before treatment: VASa=6.89 VASr=4.28; 6 months post-treatment: VASa=1.61 VASr=1.44 *significant difference within group. Steroid injection group + standard PT: before treatment: VASa=6.22 VASr=2.89; 6 months post-treatment: VASa=5.72 VASr=2.94 *non-significant change within group ** significant difference between groups	LAAG(0-100 score) RFD + standard PT group: before treatment: LAAG score: 47.22; after treatment: LAAG score=83 *significant change within group. Steroid injection group + standard PT: before treatment: LAAG score=45.67; after treatment: LAAG score=46.06 *non-significant change within group **significant difference between groups	Not assessed	None

Hölmich et al. [12] described the effects of an active training program (AT), based on improving coordination and strength of the muscles of the pelvis and hip (especially the adductor muscles), on the outcome of athletes presenting with adductor-related groin pain. This program was compared against a standard physiotherapy (PT) program consisting of laser treatment, massage, stretching, and transcutaneous electrical nerve stimulation (TENS). Hölmich et al. reported that 79% of patients completing the AT program returned to sports at the previous level of activity, with a mean time to return to sports of 18.5 weeks. Hip range of motion (ROM) was significantly improved in both groups, but it did not differ significantly between groups. However, adduction strength was significantly improved in the AT group compared to the PT group (p=0.001). Outcomes in this study were classified as excellent, good, fair, or poor, whether participants reached three, two, one, or none of the following measures: no pain at palpation of the adductor tendons and adductor insertions at the pubic bone and no pain during active adduction against resistance, no groin pain in connection with or after athletic activity in the same sport and at the same level of competition as before the onset of groin pain, and return to same sport and at the same level without groin pain. The distribution of the outcomes showed a significant difference favoring the AT group (p=0.001) with 25/34 showing good to excellent results compared to the PT group where only 10/34 showed the same outcomes.

Weir et al. [13] compared the effects of a multi-modal treatment (MMT) approach, consisting of heat followed by manual therapy and stretches, against an exercise therapy (ET) approach with the same exercises as the ones used by Hölmich et al. In this study, there were no significant differences between the groups in the percentage of athletes returning to sports (50% in the MMT treatment, 55% in the ET treatment); however, there was a significantly faster return to sports in the MMT treatment group (mean time 12.8 weeks vs 17.3 weeks). In the objective outcome assessment, there were also no significant differences between groups, with 12/22 reaching good to excellent results on the ET treatment and 14/26 on the MMT group reaching the same results. In this study, VAS scores improved in both groups (p=0.000) but there were no significant differences between groups (p=0.12). Hip ROM did not improve significantly in both groups after treatment.

Abouelnaga et al. [14] studied the effects of an active therapy program (ATP), consisting of hip and abdominal muscles, strengthening exercises, core stabilization, and balancing exercises, against a conventional treatment program (CT), consisting of heat, massage, TENS, mobilization, and stretches. This study showed a significant decrease in VAS in both groups (p=0.0001) compared to pre-treatment. There was also a significant decrease in VAS in the ATP group compared to the CT group (p=0.0001). Hip ROM showed a significant increase in both groups; however, there were no differences in internal or external rotation between groups. In this study, 65% (13/20) of the athletes in the ATP group returned to sports at previous levels compared to only 15% (3/20) in the CT group, and 75% (15/20) showed good to excellent outcomes in the ATP group compared to only 35% (7/20) in the CT group.

Schöberl et al. [18] studied the effect of a standard intensive physical rehabilitation program with or without shock wave (SW) therapy against a control group that did not participate in a rehabilitation program. The intensive program consisted of three phases: in the first phase (days 1 to 28), no sports were allowed, and athletes received physiotherapy 90 min, three times per week, consisting of lymphatic activation, blockage release in the lumbar spine and hip joints, trigger point therapy, myofascial techniques, muscular tonus release in the adductor and abdomen muscles, and mobilization of the pelvis, hip, lumbar spine, and sacrum. In the second phase (days 29 to 56), light sports were allowed (cycling, skating, and swimming) and stretches. Physiotherapy was performed as in phase 1. In the third phase (after day 56), proprioceptive exercises and football-specific training were started. Physiotherapy in this phase consisted of muscular tonus release, blockage release in the lumbar spine and hip, and trigger point therapy and was continued until one year. SW therapy was applied directly to the pubis in the treatment group, while the other group received sham shock wave therapy. In this study, primary outcomes were a reduction in pain measured by VAS score and the time to return to football defined as the time of the first competitive match. There was a significant decrease in the VAS scores in both groups starting one month after treatment (p<0.001) and a significant difference between groups at one month and three months after treatment (p<0.001). In this study, 95% (42/44) of athletes returned to sports within four months, but the return to play was significantly faster in the SW therapy group (73.2 days vs 102.6 days, p=0.048). The control group that only abstained from sports showed a much longer return to play time (240 days, p<0.001).

Moreno et al. [15] evaluated the utility of intratissue percutaneous electrolysis (EPI) in combination with active physical therapy (APT) to treat adductor groin pain (refer to the original article for a complete description of the APT program). This technique was compared against a group that underwent only APT. The outcome measures in this study were the numeric rating scale (NRS) and the patient-specific functional scale (PSFS). The NRS values were collected upon palpation of the insertion of the adductor longus (NRSpalp) and bilateral isometric contraction against resistance (NRScontr). The APT consisted of three phases. Patients progressed from phase 1 to phase 2 if they score ≤3/10 in the NRScontr and NRSpalp tests. They then progressed to phase 3 if the PSFS score was ≥8. EPI was applied during phase 1 of the APT program in two treatment sessions per week. In this study, both groups showed significant improvements in both NRS scores and PSFS (p<0.001); however, NRScontr was significantly lower in all time points in the EPI group compared to the APT-only group. NRSpalp only showed significant differences between groups at two months and four months. No differences between groups were found for the PSFS (p=0.093). The addition of EPI to phase 1 of the APT program also reduced its duration (mean 8.8 days, p=0.048).

Comin et al. [16] studied the effects of radiofrequency denervation (RFD) of the ilioinguinal nerve and ligament on groin pain. The study compared three groups: one group was treated with RFD plus standard physiotherapy (consisting of exercises for adductors, abdominals, and gluteal muscles), the second group was treated with local anesthetic plus steroid and standard physiotherapy, and the third group was a non-randomized group which had already performed previous surgery (not considered for analysis in this review). The outcome measures in this study were the VAS during activity and rest (VASa and VASr) and the London Adductor and Abdominal Groin score (LAAG) as a function measure. The VAS and LAAG were improved in the RFD group in all time points up to six months (p<0.001). In the steroid injection group, only LAAG and VASa were improved, and only at week 1. There was a significant difference between the RFD and steroid groups in the LAAG and VAS scores.

In the double-blinded RCT by Otten et al. [17], athletes were randomized to wear either zoned high compression shorts (ZHC shorts), non-zoned low compression shorts (NZLC shorts), or no compression shorts, and the effects on pain and performance were measured. The outcome measures were the NRS and performance during the Copenhagen 5-s squeeze test (CS), the Illinois agility test (IAT), and maximum shooting (ST). The hip and groin outcome score (HAGOS) was used to measure the effects on symptoms when wearing ZHC versus NZLC shorts during actual football activities. This study revealed that wearing ZHC shorts significantly reduced pain during the IAT (medium effect size) and the ST (small effect size), without negatively impacting performance, compared to wearing no compression shorts. Wearing ZHC shorts during training and matches also resulted in a small but significant reduction in pain compared to wearing the NZLC shorts. Concerning the HAGOS scores, wearing ZHC shorts during football activities significantly improved the HAGOS scores on activities of daily living symptoms (ADL) and sport and recreation subscales (medium effect sizes).

Risk of Bias Assessment

**Figure 2:**
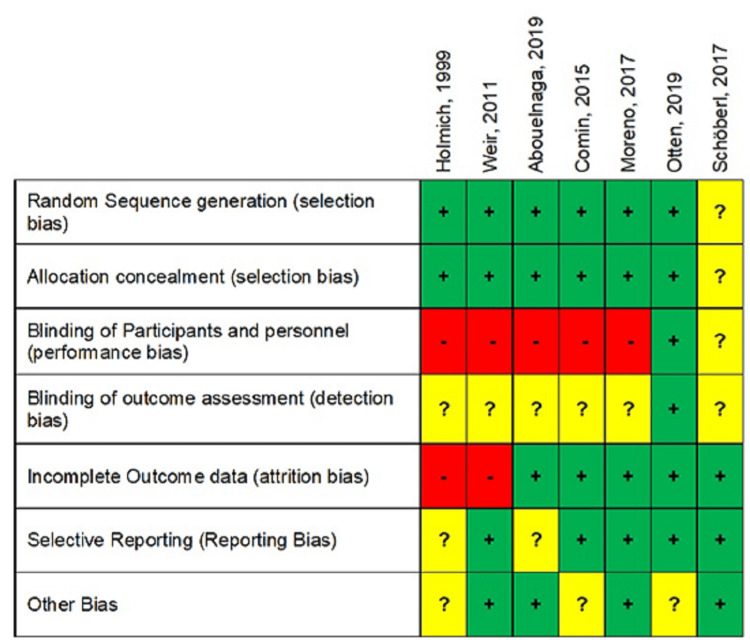
Risk of bias summary presentation

The risk of bias detailed assessment of each included study is presented in Table 4, and its summary presentation is shown in Figure 2.

**Table 4:** Risk of bias assessment AT: active training program, PT: standard physiotherapy program, LAAG: London Adductor and Abdominal Groin score

Bias/reference	Hölmich et al. (1999) [12]	Weir et al. (2011) [13]	Abouelnaga et al. (2019) [14]	Comin et al. (2013) [16]	Moreno et al. (2017) [15]	Otten et al. (2019) [17]	Schöberl et al. (2017) [18]
Random sequence generation (selection bias)	Low risk	Low risk	Low risk	Low risk	Low risk	Low risk	Unclear risk
	Comment:	Comment:	Comment:	Comment:	Comment:	Comment:	Comment:
	Patients were randomly allocated by sealed, opaque, and serially numbered envelopes to AT or PT by means of block randomization	Athletes were randomized using sealed envelopes	The randomization process was applied using the envelope method	Randomization was generated by dedicated software (StatsDirect Ltd, Cheshire, UK). This was operated by an independent individual, who did not participate in the treatment	To randomize the groups, the following tool was used: “create a blocked randomization list” (Sealed envelope ltd. 2014)	Block randomization with balanced permutations was done before data collection commenced	The study states that participants were randomized, although the method of randomization is not clearly described. The control group was not randomized
Allocation concealment (selection bias)	Low risk	Low risk	Low risk	Low risk	Low risk	Low risk	Unclear risk
	Comment:	Comment:	Comment:	Comment:	Comment:	Comment:	Comment:
	The examining physician was not involved in the randomization procedure and remained unaware of the treatment allocation	The examining physician was not involved in the randomization procedure and remained unaware of the treatment allocation	The examining physical therapist was not involved in the randomization process and remained unaware of the treatment allocation. Athletes were instructed not to reveal their treatment allocation to the physical therapist during their assessment	Randomization was generated by dedicated software. Results of randomization were presented to the proceduralist (DC) immediately prior to the procedure	Each subject included in the study was given their personal code assigning them to one of the two groups. The code was enclosed in sealed envelopes	Randomization was done using the software. The order of the test conditions was randomized in order to minimize the learning effect and the effect of fatigue	There is no clear information on how patients were allocated to the study groups
Blinding of Participants and personnel (performance bias)	High risk	High risk	High risk	High risk	High risk	Low risk	Unclear risk
	Comment:	Comment:	Comment:	Comment:	Comment:	Comment:	Comment:
	Participants and therapists could not be blinded to allocated treatment due to the nature of the interventions	Participants and therapists could not be blinded to allocated treatment due to the nature of the interventions	Participants and therapists could not be blinded to allocated treatment due to the nature of the interventions	Participants could not be blinded to allocated treatment due to the nature of the interventions	Participants could not be blinded to the allocated treatment due to the nature of the interventions	All types of shorts were identical. Participants were instructed not to tell the investigators about the current test condition in order to ensure investigator blinding	Study states that participants, physicians, and physiotherapists were blinded for the application of shock wave therapy, but the blinding method was not described
Blinding of outcome assessment (detection bias)	Unclear risk	Unclear risk	Unclear risk	Unclear risk	Unclear risk	Low risk	Unclear risk
	Comment:	Comment:	Comment:	Comment:	Comment:	Comment:	Comment:
	Subjective outcomes assessed from unblinded participants	Subjective outcomes assessed from unblinded participants	Subjective outcomes assessed from unblinded participants	Blinding of outcome assessors was not described	Blinding of outcome assessors was not described	Study was double-blinded	Blinding of outcome assessors was not described
Incomplete outcome data (attrition bias)	High risk	High risk	Low risk	Low risk	Low risk	Low risk	Low risk
	Comment:	Comment:	Comment:	Comment:	Comment:	Comment:	Comment:
	Characteristics of drop-outs were not described. A per-protocol analysis was done	Characteristics of drop-outs from the study were not described. A per-protocol analysis was done	No patient was lost before the study's completion	No patient was lost before the study's completion	Two patients lost to follow-up equally distributed between groups and reasons for the loss were correctly described	7 patients lost to follow-up correctly described Effects of the Shorts on pain and comfort during football activities were analyed using intention-to-treat analysis using a standard paired t-test.	No patient was lost before study's completion
Selective reporting (reporting bias)	Unclear risk	Low risk	Unclear risk	Low risk	Low risk	Low risk	Low risk
	Comment:	Comment:	Comment:	Comment:	Comment:	Comment:	Comment:
	Some important primary outcomes were incompletely reported in the results	All intended outcomes were reported and assessed	Some function outcomes such as RTP were incompletely reported. RTP time an important outcome was not reported	All intended outcomes were reported and assessed	All intended outcomes were reported and assessed	All intended outcomes were reported and assessed	All intended outcomes were reported and assessed
Other bias	Unclear risk	Low risk	Low risk	Unclear risk	Low risk	Unclear risk	Low risk
	Comment:	Comment:	Comment:	Comment:	Comment:	Comment:	Comment:
	Imbalance between groups in the baseline incidence of bilateral groin	No other sources of bias were found	No other sources of bias were found	LAAG questionnaire has not been validated	No other source of bias was identified	Carry-over effect cannot be excluded	No other source of bias was identified

None of the studies was classified as low risk of bias in all of the domains. However, some considerations are important to make. Since all studies were measuring subjective pain outcomes, blinding of participants was essential. However, physiotherapy and minimally invasive interventions make the introduction of performance bias unavoidable since the blinding of participants and personnel to the treatment performed is impossible. As such, most studies were classified as high risk of bias in this domain. Only one double-blinded randomized trial was classified as low risk [17] because blinding of the intervention being tested was possible (ZHC shorts and NZLC shorts were identical and randomly assigned). One double-blinded RCT [18] was scored as an unclear risk of performance bias as the methodology of blinding participants and personnel was not clearly described. In relation to selection bias, most of the studies were classified as low risk, as the process of randomization and allocation concealment were clearly described. Only one study was classified as unclear risk [18] because the method of randomization was not clearly stated in the methodology. Detection bias was generally classified as unclear risk as methods of blinding outcome assessors were not clearly stated in most studies, and some required the collection of subjective outcomes from participants, which were not blinded, introducing a potential risk of knowledge of which intervention was received. Overall, most studies had a low risk of attrition bias, as reasons for losing participants to follow-up were clearly described, and the intention to treat analysis when appropriate was performed. In this respect, only two studies were classified as high risk of attrition bias, as the reasons for the dropouts from the study were not described and a per-protocol analysis was performed. For the domains of reporting bias and other sources of bias, overall studies were classified as low risk.

In conclusion, two studies were overall classified as high risk of bias and five were classified as unclear risk of bias. The authors of this review consider that performance bias will always be present in these kinds of studies. Therefore, a lower weight needs to be attributed to this domain when classifying the overall risk of bias across studies. Overall, the risk of bias across studies was considered to be an unclear risk of bias.

Certainty of Evidence

Because pooling of data was impossible to carry out, due to outcome measures being different across studies and differently reported, a meta-analysis was not possible to perform to assess the effect size of the treatment. However, the authors of this review decided to provide a tentative assessment of the certainty of the available evidence adopting an approach previously described by Murad et al. [11]. This method applied the principles of the GRADE approach [19] to judge the certainty of evidence when a single quantitative estimate size of the effect is not possible to ascertain. Table 5 illustrates how the authors used this approach to rate the certainty of evidence and Table 6 represents the summary of findings.

**Table 5:** Certainty of evidence

GRADE domain	Judgment	Concerns about certainty domains
Risk of bias	Most of the studies had a low risk of bias in sequence generation and allocation concealment (6 out of 7). Two studies had a high risk of attrition bias, and five studies had a high risk of performance bias, although the nature of the studies made it impossible to avoid. All studies had at least one domain classified as uncertain risk. Therefore, we classified articles as having an uncertain risk of bias.	Serious
Imprecision	The total number of trial participants was 345. Some trials reported small effects or no significant differences between groups on some measures. However, all studies reported significant outcomes when testing nonsurgical interventions. We judged the evidence to have no serious imprecision.	Not serious
Inconsistency	Most studies consistently reported the positive effects of nonsurgical interventions. However, there was significant variability in the populations included, interventions provided, and outcomes measured potentially leading to heterogeneity.	Serious
Indirectness	The evidence is applicable to our relevant question. The outcomes were assessed using different scales in different trials. We judged the evidence to have no serious indirectness.	Not serious
Publication bias	We have not detected any source of publication bias. A systematic search for studies was performed.	Not serious

**Table 6:** Summary of findings (certainty of evidence) Certainty of evidence: high, ⊕⊕⊕⊕; moderate, ⊕⊕⊕O; low, ⊕⊕OO; very low, ⊕OOO

Outcome	Effect	Nº participants	Certainty of evidence
Reduction of pain and return to full sports after nonsurgical interventions using various measures.	Most studies showed significant reductions in pain and return to sports at previous levels after treatment.	345 (7 RCTs)	LOW* ⊕⊕OO *(due to risk of bias and inconsistency)

Discussion

The purpose of this systematic review was to evaluate the effectiveness of nonsurgical interventions in the treatment of groin pain. The literature search revealed a scarcity of randomized controlled trials. The available studies were relatively well designed but had some limitations (some inherent to the nature of the tested interventions) which were possible confounding sources of bias. As a result, the certainty of evidence was assessed to be low. Nonetheless, the analysis of the outcome data revealed that in athletes with groin pain, nonsurgical interventions can lead to significant favorable outcomes concerning pain reduction and return to sports. The body of evidence in this review suggests that an active exercise program is effective in the reduction of groin pain and should be a staple component in any conservative treatment for groin pain. A multimodal treatment, as reported by Weir et al., allowed a faster return to sports compared to active physical therapy; however, there were no differences in the pain measures, percentage of athletes returning to sports, or objective outcome assessment compared to the active treatment group.

The addition of shock wave (SW) therapy to an intensive rehabilitation program allowed a faster return to sports and a faster reduction of pain. Interestingly, the group of patients that performed SW therapy had the fastest time to return to sports, compared to the other studies that reported the same outcome (mean 10.5 weeks vs 12.8 and 18.5 weeks [12,13]). Although this was a double-blinded RCT, more studies are needed to confirm this result as there were some doubts in the methodology of the study which was not clearly described in the article.

When conservative approaches fail to improve symptoms, some minimally invasive procedures can be performed. In this respect, the available evidence demonstrated that RFD of the ilioinguinal nerve and inguinal ligament, when combined with physiotherapy, could significantly reduce groin pain compared to steroid plus anesthetic injection and physiotherapy. The magnitude of the reduction of pain outcome measures after RFD was similar to the ones described in other studies testing other conservative interventions. This was a single study and the results of this procedure should be subject to more investigation.

Another minimally invasive and recently described procedure for groin pain is intratissue percutaneous electrolysis (EPI). In the only RCT evaluating the efficacy of this procedure, there was a faster and greater reduction of pain in the group receiving the treatment when combined with standard physiotherapy. This was a well-designed study with few limitations and no conflicts of interest. The treatment was well tolerated and there were no adverse events; however, one patient dropped out due to fear of needles, which makes the procedure undesired by some patients. In addition, this was the only RCT on the subject and more data is needed to draw clear conclusions.

In the study by Otten et al., wearing high-compression shorts significantly reduced pain in some performance measures and improved symptoms. This study was a double-blinded RCT, with a low risk of bias, providing good evidence that wearing high-compression shorts can be useful for the control of ongoing groin pain for at least a one-month duration. In addition, the intervention had no real contraindications and didn’t affect the athlete’s performance.

Although the certainty of evidence available on this subject was graded as low when considering the strength of recommendation for a certain treatment, other factors need to be considered, and not always the intervention with a high grade of certainty is qualified as a strong recommendation. Some of the factors that may impact this assessment are the cost-effectiveness of the treatment, the balance of desirable and undesirable effects, and the patient’s preference. The conservative approaches previously described are relatively cheap, without significant health risks, and usually desired by the patient as an initial approach, and as such, recommending nonsurgical treatments for the management of groin pain takes priority over surgical ones. In the author’s opinion, despite the low quality of the available evidence when considering the mentioned factors, the strength of recommendation for nonsurgical treatments in the management of groin pain in athletes should be classified as strong as the benefits significantly outweigh the risks of the treatment. However, no clear recommendations can be made about the preference for one nonsurgical intervention over another as more high-quality studies are needed to reach definitive conclusions. Nonetheless, the current evidence shows that active physical therapy seems to constitute the base of a successful treatment program.

Limitations

This review has some limitations, the first being that only a few randomized controlled trials were available and included in the systematic review. Furthermore, by discarding all the lower-quality studies, potentially useful information may have been lost on the subject.

The second limitation is that the authors were not able to pool the data for quantitative analysis due to the obvious heterogeneity of interventions, outcome measures, and inconsistencies in methodology. Unfortunately, this led the authors to provide only a narrative description of outcomes. The third limitation is that the method of ascertaining the certainty of evidence in the absence of pooled data, although based on the GRADE approach, has not yet been validated. However, we feel this approach provided an accurate grade of the quality of the evidence.

## Conclusions

More RCTs evaluating the efficacy of nonsurgical interventions for the management of groin pain are needed. The current evidence demonstrates a significant positive effect of active physiotherapy programs, and these should probably be the initial approach to treatment. Minimally invasive procedures, such as the ones described in our review, should probably be tried before more complex interventions are adopted, such as surgery.

New RCT studies should try to minimize bias by increasing the quality of the study’s design. However, the nature of some interventions in the field doesn’t allow participants and personnel to be blinded, and, although this can introduce potential bias, performance bias should not constitute a significant criterion to rate lower the quality of otherwise high-quality studies. Newer studies should use comparable single outcome measures when evaluating intervention’s effects, such as VAS, NRS, RTP rate, and RTP time, and standardize the reporting of data so that a meta-analysis can be performed. Function scores are also important to analyze and studies should use validated questionnaires such as the HAGOS.
